# Interstitial Brachytherapy for Hepatocellular Carcinoma: Analysis of Prognostic Factors for Overall Survival and Progression-Free Survival and Application of a Risk Stratification Model

**DOI:** 10.1159/000531732

**Published:** 2023-06-29

**Authors:** Maximilian Thormann, Franziska Heitmann, Vanessa Wrobel, Constanze Heinze, Christine March, Peter Hass, Robert Damm, Alexey Surov, Maciej Pech, Jazan Omari

**Affiliations:** ^a^Department of Radiology and Nuclear Medicine, University Hospital Magdeburg, Magdeburg, Germany; ^b^Department of Radiation Oncology, University Hospital Magdeburg, Magdeburg, Germany; ^c^Department of Radiology, Neuroradiology and Nuclear Medicine, Johannes Wesling University Hospital, Ruhr University Bochum, Minden, Germany

**Keywords:** Hepatocellular carcinoma, Interstitial brachytherapy, Low skeletal muscle mass, Overall survival, Progression-free survival, Prognostic model

## Abstract

**Introduction:**

Interstitial brachytherapy (iBT) is an effective treatment for hepatocellular carcinoma (HCC). Identification of prognostic factors is pivotal for patient selection and treatment efficacy. This study aimed to assess the impact of low skeletal muscle mass (LSMM) on overall survival (OS) and progression-free survival (PFS) of iBT in patients with HCC.

**Methods:**

For this single-center study, we retrospectively identified 77 patients with HCC who underwent iBT between 2011 and 2018. Follow-up visits were recorded until 2020. The psoas muscle area, psoas muscle index, psoas muscle density (MD), and the skeletal muscle gauge were assessed on the L3 level on pre-treatment cross-sectional CT scans.

**Results:**

Median OS was 37 months. 42 patients (54.5%) had LSMM. An AFP level of >400 ng/ml (hazard ratio [HR] 5.705, 95% confidence interval [CI]: 2.228–14.606, *p* = 0.001), BCLC stage (HR 3.230, 95% CI: 0.972–10.735, *p* = 0.026), and LSMM (HR 3.365, 95% CI: 1.490–7.596, *p* = 0.002) showed a relevant association with OS. Weighted hazard ratios were used to form a predictive risk stratification model with three groups: patients with low risk (median OS 62 months), intermediate risk (median OS 31 months), and high risk (median OS 9 months). The model showed a good prediction of 1-year mortality, with an AUC of 0.71. Higher MD was associated with better PFS (HR 0.920, 95% CI: 0.881–0.962, *p* < 0.001).

**Conclusion:**

In patients undergoing iBT for HCC, LSMM is associated with worse OS. A risk stratification model based on LSMM, AFP >400 ng/mL, and BCLC stage successfully predicted patient mortality. The model may support and enhance patient selection.

## Introduction

Hepatocellular carcinoma (HCC) accounts for approximately 90% of primary liver cancer. It is one of the most frequently diagnosed cancer types worldwide and a leading cause of cancer-related deaths [1]. The majority of cases in the Western population develop in cirrhotic livers. Treatment depends on the stage of the cancer and various variables, including liver function, performance status, and the number and size of lesions [2]. Image-guided minimally invasive interventions have an integral role in the treatment of HCC [3]. For patients who are not suitable for surgical resection, local ablation can be performed either as a curative attempt or to reduce the tumor burden [4]. Common ablation techniques include radiofrequency ablation (RFA), microwave ablation, and intra-arterial therapies such as transarterial chemoembolization (TACE) [5]. An alternative to these techniques is interstitial brachytherapy (iBT), which has shown low rates of local recurrence [6−8]. Compared to thermal ablation techniques, iBT offers the advantage of not being restricted by tumor size, heat sink effects, or portal vein thrombosis [9].

iBT has only been established for several years and an identification of optimal stratification parameters is still lacking. There is increasing evidence that low skeletal muscle mass (LSMM), used as a proxy for sarcopenia, is an important factor associated with adverse clinical outcomes in various oncologic diseases [10−14]. For local ablation in liver tumors, studies have found an association between sarcopenia and overall survival (OS) in patients undergoing RFA [15−17]. No data are yet available for microwave ablation or iBT.

The aim of this study was to assess the influence of pre-treatment LSMM on OS for patients undergoing iBT for HCC. We utilized the psoas muscle index (PMI) as a marker of LSMM. In addition, we analyzed the impact of psoas muscle density and the skeletal muscle gauge (SMG).

## Methods

### Study Design

From our internal database, we retrospectively identified 107 patients with HCC who underwent iBT at our institution from 2011 to 2018. The indication for therapy was determined by an interdisciplinary tumor board. Generally, percutaneous iBT was recommended for HCC patients with the following characteristics: (1) BCLC stage 0-C, (2) preserved liver function Child-Pugh A or B, (3) ECOG 0–1, (4) up to three lesions. There was no upper limit placed on the tumor diameter. All patients were followed-up at our department for scheduled visits every 3–6 months after therapy until 2020. In cases of tumor progression or local recurrence, the most appropriate therapy − systemic therapy, another brachytherapy, or a combination of both − was selected for each patient based on interdisciplinary consensus.

#### Inclusion criteria were as follows:

- Confirmed diagnosis of HCC.
- Available CT scan including the psoas muscle on the level of L3 within 3 months before treatment.
- Available clinical data regarding OS.

#### Exclusion criteria were as follows:

- Missing pre-treatment CT images.
- Strong motion or metal artifacts in CT scans.
- Missing clinical data.
- Missing body height.

**Fig. 1:**
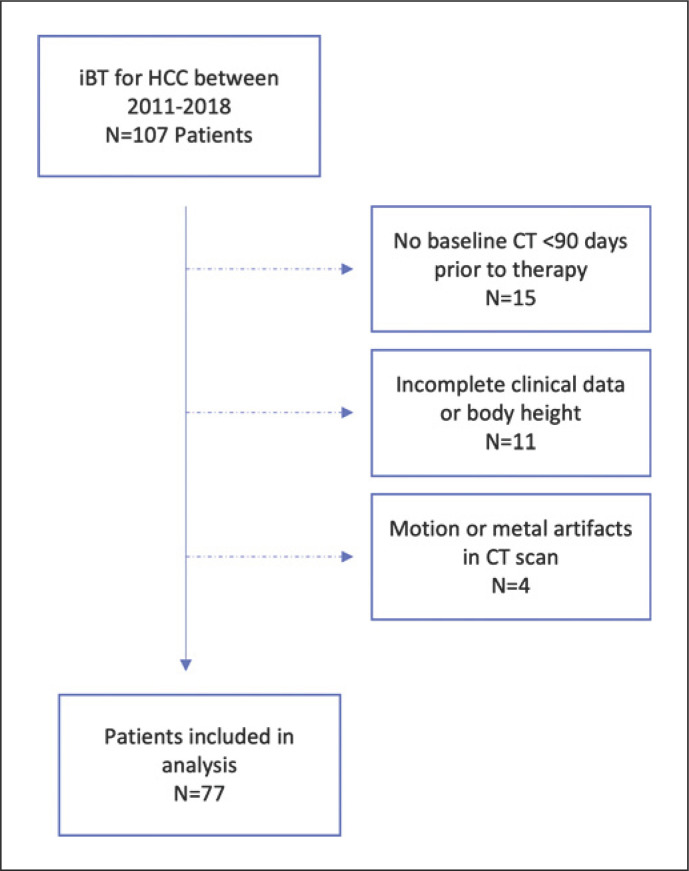
Patient selection algorithm.

The patient selection algorithm is illustrated in Figure 1. The study was approved by the Ethics Committee of the University of Magdeburg, Medical Faculty (No. 145/21). Due to the retrospective design of the analysis, patient consent was waived in accordance with local and national guidelines.

### Brachytherapy Procedure

The procedure of percutaneous brachytherapy has been reported previously [7, 18, 19]. In short, after analgosedation of the patient, irradiation catheters were placed in the target lesions using CT-fluoroscopy or MR guidance by Seldinger's technique. In general, one catheter was inserted per 1–2 cm tumor diameter, depending on tumor shape and location. Treatment was performed in a single-fraction irradiation in afterloading technique using an iridium 192 source. For HCC, a target dose of 15 Gy was applied. After treatment, the puncture tracks were closed with Gelfoam.

### Image Analysis

All CT scans were performed on a multidetector CT scanner (Canon Aquilion Prime, Otawara, Japan) with patients in the supine position. For image analysis, we referred to the last available abdominal CT scan within 3 months prior to iBT. All CT measurements were performed in consensus by two experienced radiologists (MT and AS) with 4 and 16 years of experience in abdominal imaging, respectively, who were blinded to the clinical course of patients.

**Fig. 2:**
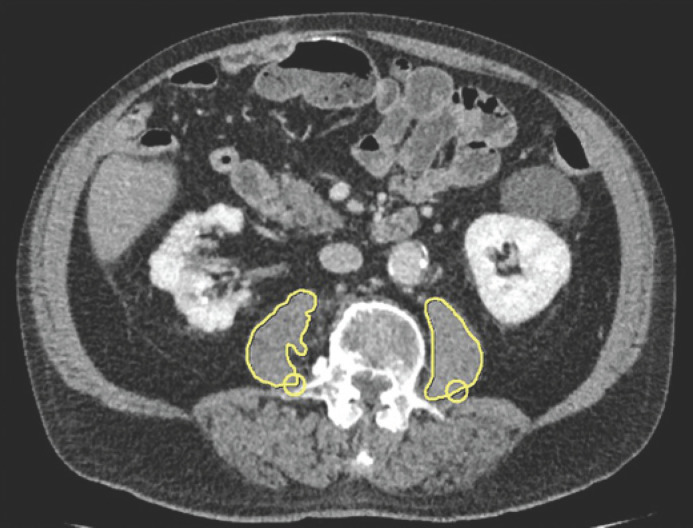
Exemplary illustration of body composition measurements. A line was drawn along the contours of the psoas muscles on both sides. The bilateral areas were added to obtain the psoas muscle area (PMA).

We applied the PMI for detection of LSMM and as a proxy for sarcopenia. PMI is calculated by dividing the sum of the bilateral psoas muscle area (PMA) by the height squared in cm. Measurements were performed manually on axial images at the mid-L3 level in the soft tissue window (45–250 HU) on a dedicated workstation (Infinitt PACS, Version 3.0, Infinitt Healthcare, Korea). Two radiologists manually drew a line along the contours of the psoas muscles on both sides and the bilateral areas as calculated by the software were added to obtain the PMA (Fig. 2). LSMM was defined as a PMI of <5.40 cm^2^/m^2^ for males and <3.56 cm^2^/m^2^ for females [20]. The mean psoas muscle density was calculated from the values from both sides. SMG combines the PMI and muscle density and has been associated with outcomes in breast cancer patients [21, 22]. It is calculated by multiplying mean psoas muscle density with PMI as was previously reported [22]. SMG units are cm^2^ × HU/m^2^ but are reported as arbitrary units for simplicity.

### Statistical Analysis

We used SPSS version 26 for statistical analysis. For continuous variables, mean and standard deviation as well as median and interquartile range were calculated. To assess the impact of psoas muscle composition on survival, we used a univariate cox regression analysis. For factors with a significance value of *p* < 0.05 adjusted prognostic ability to predict OS was further assessed using a multivariate Cox regression with forward selection. Hazard ratios (HRs) are presented together with 95% confidence intervals (95% CIs). Discrimination values regarding survival were analyzed applying the Kaplan-Meier method and log-rank test. For regression analysis, BCLC was treated as a dummy variable (0/A = 0, B/C = 1).

Results from the multivariate regression analysis were weighted and used to calculate a predictive risk model with low-risk, intermediate-risk, and high-risk groups. The HR of each parameter was taken from the multivariate regression model and then normalized into weighted scores. We used area under the receiver-operating characteristic curve to test the model's ability in predicting survival.

## Results

### Characteristics of Cohort

Of the 107 patients in our database, 77 were included in our analysis. A comparison of characteristics of included and excluded patients is shown in Table 1. Of the included cohort, 67 patients were male, 10 were female. By definition of PMI, 42 patients (54.5%) were suffering from LSMM. Median time between analyzed CT scans and treatment was 5 days (range 0–90 days). 63 interventions were performed by CT guidance and 14 by MRI guidance. The median applied dose was 15.9 Gy. Liver cirrhosis was present in 69 patients. Three patients had extrahepatic tumor manifestation, one with a lung metastasis and two with adrenal gland metastases. Median AFP was 12 ng/mL. Due to recurrence of HCC, 27 patients received systemic therapy after iBT. 16 patients had a lesion size of >5 cm. There were no significant differences in baseline characteristics for the two groups. Non-LSMM patients had a slight tendency toward smaller lesions. Patient baseline characteristics are summarized in Table 2.

### Progression-Free Survival

Median progression-free survival (PFS) was 2.24 years. There was no relevant association between PMI (HR 0.946, 95% CI: 0.811–1.103, *p* = 0.476), PMA (HR 0.982, 95% CI: 0.928–1.039, *p* = 0.531), SMG (HR 0.997, 95% CI: 0.994–1.00, *p* = 0.068), or LSMM (HR 1.208, 95% CI: 0.733–1.989, *p* = 0.458) and PFS. Muscle density showed a relevant influence on PFS (HR 0.920, 95% CI: 0.881–0.962, *p* < 0.001).

### Overall Survival

**Fig. 3:**
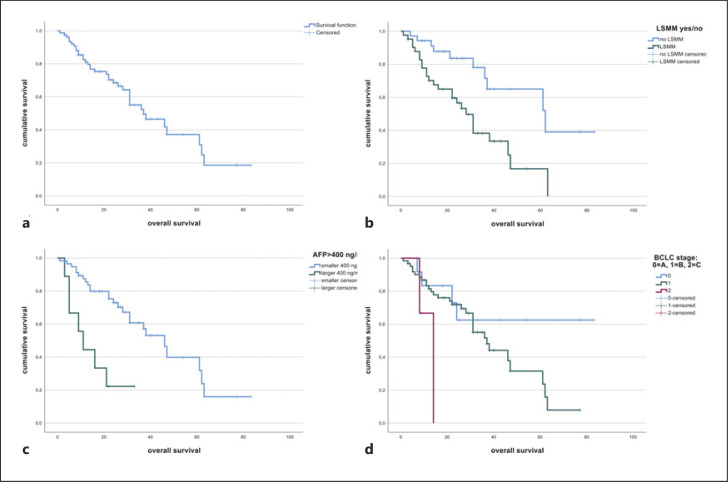
Kaplan-Meier curve for OS in the overall cohort (**a**) in the LSMM and non-LSMM group (**b**), for AFP >400 ng/mL and <400 ng/mL (**c**), and for BCLC stages (**d**).

Median OS was 37 months (SD 8 months) (Fig. 3a). In a univariate cox regression analysis, PMI (HR 0.806, 95% CI: 0.657–0.989, *p* = 0.039), muscle density (HR 0.948, 95% CI: 0.906–0.991, *p* = 0.019), and SMG (HR 0.994, 95% CI: 0.991–0.998, *p* = 0.005) showed a relevant influence on OS. Comparing groups with and without LSMM, LSMM was significantly associated with OS (HR 3.085, 95% CI: 1.464–6.504, *p* = 0.003). Of the clinical and laboratory variables, BCLC stage (HR 3.397, 95% CI: 1.564–7.378, *p* = 0.002) and AFP >400 ng/mL (HR 4.244, 95% CI: 1.745–10.322, *p* = 0.001) were associated with OS (Table 3). Prior systemic therapy did not show a relevant influence on OS (*p* = 0.378). There was no significant association of systemic therapy after iBT with survival.

In multivariate regression analysis using forward selection, AFP >400 ng/mL (HR 5.705, 95% CI: 2.228–14.606, *p* = 0.001), BCLC stage (HR 3.230, 95% CI: 0.972–10.735, *p* = 0.026), and LSMM (HR 3.365, 95% CI: 1.490–7.596, *p* = 0.002) showed a relevant influence on survival (Table 3). Patients with LSMM had a median OS of 28 months (SD 3.2 months), while patients without LSMM showed a median OS of 62 months (SD 15.1 months). The log-rank test revealed a significant difference between the two groups (*p* = 0.002) (Fig. 3b).

Patients with AFP >400 ng/mL had a median OS of 11 months (SD 3.0 months), patients with an AFP >400 ng/mL had an OS of 46 months (SD 5.5 months). The log-rank test revealed a significant difference between the two groups (*p* < 0.001) (Fig. 3c).

For BCLC, a higher stage was associated with decreased OS, with stage B showing a survival time of 37 months (SD 5.2 months) and stage C of 14 months (SD 0 months). Of the 3 patients with BCLC stage C, 2 died during the observation period (Fig. 3d).

### Risk Stratification

The risk stratification model was derived from the HRs generated by the multivariate regression. BCLC was treated as a dummy variable (0/A = 0, B/C = 1). The smallest HR was found to be 3.230 for BCLC stage. BCLC stage received a weight of 1.0. LSMM received a rounded weight of 1.0. AFP >400 ng/mL received a rounded weight of 2.0.

**Fig. 4:**
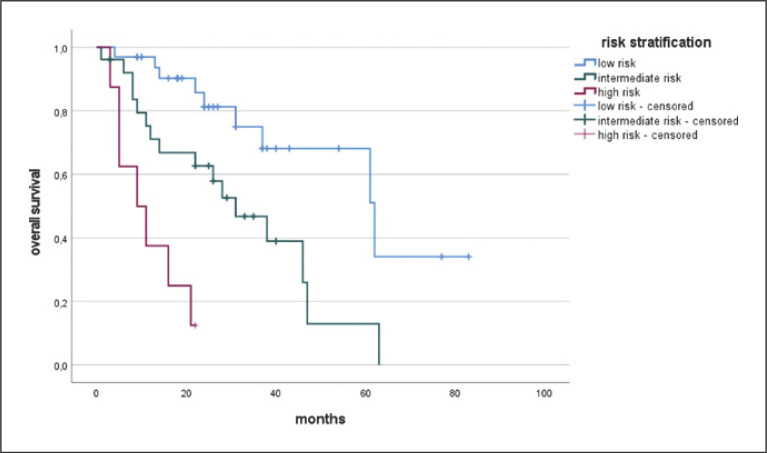
Kaplan-Meier curves for OS of HCC patients undergoing iBT. Stratification by risk groups. Median OS was 62 months for the low risk, 31 months for the intermediate risk, and 9 months for the high-risk group. The model was confirmed by ROC analysis and 1-year survival with an area under the curve (AUC) of 0.711 (*p* = 0.01).

This generated a prognostic model with a minimum of 0 and a maximum of 4 points. The range was divided into three equal sections with three risk groups: low-, intermediate-, and high-risk groups. The low-risk group had a range of 0–1.3 points, the intermediate group >1.3–2.7, and the high-risk group >2.7–4 points. Patients in the low-risk group had a median OS of 62 months (SD 13.1 month), while those in the intermediate- and high-risk group had a median OS of 31 months (SD 6.9 months) and 9 months (SD 4.2 months), respectively (Fig. 4). The log-rank test revealed a significant difference between the groups (*p* < 0.001). The model was confirmed by ROC analysis and 1-year survival with an area under the curve (AUC) of 0.711 (*p* = 0.01).

## Discussion

Our retrospective study aimed to evaluate the influence of LSMM on OS in patients undergoing iBT for HCC. We found that both high AFP, BCLC stage, and LSMM were significantly associated with worse OS. Additionally, we found a weak but relevant association between higher muscle density and better PFS. To the best of our knowledge, this is the first study evaluating the impact of LSMM on patients undergoing iBT for HCC.

Local ablative treatment techniques have emerged as promising strategies for HCC. iBT has been shown to be a safe and effective treatment option for liver lesions in early-stage HCC [23, 24]. Patients referred to iBT usually are present with larger lesions or those not suitable to surgical resection or other local treatments. iBT can be applied to HCC lesions not accessible to thermal ablation methods. Studies have shown that tumor size and radiation dose affect the rate of local recurrence in iBT in the liver, while age and comorbidities do not have a significant impact [8, 25, 26].

LSMM is a common finding in patients with HCC. A recent meta-analysis reported that approximately 39% of patients had LSMM, which was associated with worse OS and lower recurrence-free survival (RFS) [27]. In our cohort, the rate of patients with LSMM was somewhat higher. This could be attributed to the high number of cirrhotic patients in our analysis (89.6%) and the significant proportion of alcoholic cirrhosis cases (41.6%). LSMM is highly prevalent in liver cirrhosis and may be even more common in cases of alcoholic cirrhosis [28]. Malnutrition is a frequent complication in cirrhotic patients [29]. Moreover, liver cirrhosis leads to changes in body composition, including an increase in extracellular fluids and a decrease in muscle and adipose tissue [29]. These changes result in alterations in cytokine and hormonal pathways, as well as impaired skeletal muscle utilization of amino acids as an energy source [30].

Numerous studies have shown the impact of sarcopenia on outcomes under various oncologic conditions. Sarcopenic patients with lung cancer, ovarian cancer, and gastrointestinal tumors have exhibited reduced OS, increased post-operative complications, and increased dose-limiting toxicity from systemic therapies [31−37]. Although different techniques were used to measure LSMM across these cohorts, studies have demonstrated a good correlation between PMI and total muscle area [38].

The existing literature on the influence of pre-treatment LSMM on outcomes after local ablation for liver malignancies remains limited. Our findings align with other studies investigating LSMM in the context of local ablation treatments. For instance, a study by Yuri et al. [16] using PMI reported that LSMM was associated with reduced OS but not with RFS in patients undergoing RFA for HCC. The cut-off values for LSMM were determined using receiver-operating characteristics. Similar outcomes were observed in a study by Yeh et al. [17] which investigated pre-treatment LSMM in early-stage HCC. Two further studies, one with a Japanese cohort [39] and one with an Egyptian cohort [15] found an adverse impact of LSMM on survival in HCC patients undergoing RFA. Only one study reported an association between LSMM and RFS [39].

In our study, patients with higher muscle density experienced better PFS, while muscle density did not show a significant association with OS. These findings are consistent with previous studies involving HCC patients. For example, Li et al. [40] found an influence of muscle density on improved PFS in patients undergoing TACE.

Our derived predictive risk stratification model shows promise as a tool for enhanced patient selection. The model indicates that the criteria of AFP >400 ng/mL, BCLC stage, and LSMM are the most predominant predictors of OS after iBT, with an AUC for 1-year survival of 0.711. Our model confirms the prognostic capacity of AFP and BCLC stage and introduces LSMM as a still underweighted factor. Other studies have also reported an association between high AFP levels and reduced OS in patients undergoing TACE and RFA [41, 42].

While LSMM is one of the three parameters influencing survival in our cohort, it is the only parameter that can be directly influenced before therapy. Our data indicate that screening for sarcopenia is pivotal before treatment as body composition parameters influence patient outcome independent of treatment success. This screening not only aids in treatment decisions but also calls for early interventions to improve patient outcomes. Multimodal interventions, such as physical exercise and nutritional supplementation can improve muscle function and prevent the loss of muscle mass [43−45]. While there is limited evidence that physical exercise may reverse the condition of LSMM, studies suggest that the rate of muscle loss can be reduced [46, 47]. Ohara et al. [48] evaluated the impact of L-carnitin supplementation in cirrhotic patients by changes in PMI per month. They found that the loss of skeletal muscle mass was significantly lower in patients receiving l-carnitine compared to the control group. Additionally, ammonia levels were lower in the intervention group. Screening for sarcopenia using pre-treatment CT imaging is easily integrated into clinical routines, and reports should include measurements of skeletal muscle.

There are several limitations to our study. It was a retrospective analysis of a single-center cohort. Only patients who received an abdominal CT scan within 3 months before therapy were included. We applied PMI as a marker for LSMM, the influence of other markers such as skeletal muscle index remains yet to be investigated. We chose pre-defined cut-off values for LSMM, rather than deriving them from our dataset. We think that this makes our results more reproducible. Given the relatively small sample size of our cohort, studies with larger patient numbers will be needed to confirm our results and individualize patient treatments.

In conclusion, our study demonstrates a significant association between LSMM and OS in patients undergoing iBT for HCC. Furthermore, a risk stratification model based on LSMM, AFP >400 ng/mL, and BCLC stage successfully predicted patient mortality. If validated in larger cohorts, this model may support and enhance patient selection. Higher muscle density is associated with better PFS.

## Statement of Ethics

The study was approved by the local Ethics Committee. Due to the retrospective design of the analysis, written patient consent was waived in accordance with local and national guidelines. The need for informed consent was waived by the Ethics Committee University of Magdeburg, Medical Faculty, Nr. 145/21.

## Conflict of Interest Statement

The authors have no conflicts of interest to declare.

## Funding Sources

The authors declare that no funding was received.

## Author Contributions

Maximilian Thormann: study design, data acquisition, data analysis drafting and revision, and final approval; Franziska Heitmann and Vanessa Wrobel: data acquisition, data analysis, and revision; Constanze Heinze, Christine March: data analysis and revision;Peter Hass, Robert Damm, and Maciej Pech: data analysis and interpretation and revision; Alexey Surov: study design, data analysis and interpretation, and drafting and revision; Jazan Omari: study conception and design, data interpretation, and drafting and revision.

## Figures and Tables

**Table 1:** Comparison of baseline characteristics of included and excluded patients

Variables	Included (*n* = 77)	Excluded (*n* = 30)	*p* value
Age, years	68 (39–87)	73 (55–90)	0.059
Sex (male), *n*	67	29	0.176
BMI, kg/m^2^	24.26	n/a^1^	−
Cirrhosis, *n*	69	25	0.510
Alcoholic cirrhosis	32	9	0.376
NASH	21	4	0.202
Viral hepatitis	5	5	0.139
Prior systemic therapy, *n*	52	18	0.502
Number of lesions at baseline, *n* (median)	1.0	1.0	0.012
Total number of lesions in obs. period, *n* (median)	2.3	2.0	0.178
Number of brachytherapies, *n* (median)	1.0	1.0	0.762
Multifocal lesions, *n*	48	14	0.191
Extrahepatic spread, *n*	4	0	1.000
Systemic therapy after brachytherapy, *n*	27	7	0.246
Child-Pugh score (median), pts	5	5	0.193
Bilirubin, µmol/L (median)	10.1	10.9	0.527
AFP, ng/mL (median)	12	7.9	0.663
<10	40.3%	56.7%	
10–399	36.4%	20.0%	
>400	10.4%	16.7%	
Portal hypertension, *n*	14	8	1.00
Portal vein thrombosis, *n*	17	2	0.090
Size, *n*			0.728
<2 cm	24	10	
2–5 cm	36	12	
>5 cm	17	8	
Dose, Gy (median)	15.9	16.1	0.061
Irradiation time, s (median)	1,435.9	1,170.5	0.023
PMA, cm^2^ (median)	15.68	18.68^2^	0.074
PMI, kg/m^2^ (median)	5.18	n/a^1^	−
Muscle density, HU (median)	47.50	41.0	0.183
SMG, AU (median)	251.18	n/a^1^	−
LSMM, *n* (%)	42 (54.5)	n/a^1^	−

BMI, body mass index; LSMM, low skeletal muscle mass; PMA, psoas muscle area; PMI, psoas muscle index; SMG, skeletal muscle gauge; AFP, alpha-fetoprotein.

1 Not enough clinical data available.

2 Data available only for 7 patients.

**Table 2:** Patient baseline characteristics

Variables	Total (*n* = 77)	LSMM (*n* = 42)	Non-LSMM (*n* = 35)	*p* value
Age, years	68 (39–87)	72 (39–87)	66 (54–84)	0.572
Sex (male), *n*	67	37	30	1.00
BMI, kg/m^2^	24.26	23.85	26.18	0.078
Cirrhosis, *n*	69	37	32	0.721
Alcoholic cirrhosis	32	18	14	0.821
NASH	21	13	8	0.454
Viral hepatitis	5	4	1	0.369
Prior systemic therapy, *n*	52	28	24	1.00
Number of lesions at baseline (median), *n*	1.0 (1–6)	1.0 (1–4)	1.0 (1–6)	0.570
Total number of lesions in obs. period (median), *n*	2.0 (1–12)	2.0 (1.7)	3.0 (1–12)	0.015
Number of brachytherapies, *n*	1.0 (1–6)	1.0 (1–5)	2.0 (1–6)	0.283
Multifocal lesions, *n*	48	23	25	0.161
Extrahepatic spread, *n*	4	3	1	1.00
Child-Pugh score (median), pts	5	5	5	0.523
Bilirubin, µmol/L (median)	10.1	9.9	12.0	0.845
AFP, ng/mL (median)	12	12.0	13.0	0.175
<10	52.2%	56.8%	46.7%	
10–399	34.3%	27.0%	43.3%	
>400	13.4%	16.2%	10%	
Portal hypertension, *n*	14	8	6	1.00
Portal vein thrombosis, *n*	17	9	8	1.00
Size, *n*				
<2 cm	24	9	15	0.049
2–5 cm	36	22	14	0.258
>5 cm	17	10	6	0.057
Dose, Gy (median)	15.9	15.9	15.9	0.649
Irradiation time, s (median)	1435.9	1457.8	1361.1	0.794
Systemic therapy after brachytherapy, *n*	27	18	9	0.120
PMA, cm^2^ (median)	15.68	14.03	19.75	<0.001
PMI, kg/cm^2^ (median)	5.18	4.55	6.12	<0.001
Muscle density, HU (median)	47.50	45.5	49	0.009
SMG, AU (median)	251.18	198.57	322.58	<0.001
LSMM, *n* (%)	42 (54.5)			

BMI, body mass index; LSMM, low skeletal muscle mass; PMA, psoas muscle area; PMI, psoas muscle index; SMG, skeletal muscle gauge; AFP, alpha-fetoprotein.

**Table 3:** Regression analysis for OS

	Univariate			Multivariate		
	HR	CI	*p* value	HR	CI	*p* value
PMA	0.944	0.880–1.011	0.101			
PMI	0.806	0.657–0.989	0.039			
Muscle density	0.948	0.906–0.991	0.019			
SMG	0.994	0.991–0.998	0.005			
BMI	1.001	0.926–1.018	0.982			
Histology	0.901	0.757–1.073	0.241			
Sex	0.874	0.338–2.258	0.780			
Prior systemic treatment	1.422	0.650–3.111	0.378			
Number of lesions at baseline	1.222	0.918–1.627	0.169			
Total number of lesions in obs. period	0.9323	0.817–1.063	0.292			
Number of brachytherapies	0.765	0.545–1.073	0.121			
Multifocal lesions	1.455	0.715–2.960	0.301			
Extrahepatic metastases	2.240	0.923–5.434	0.075			
Systemic therapy after brachytherapy	1.098	0.556–2.169	0.787			
BCLC stage^1^	3.397	1.564–7.378	0.002	3.230	0.972–10.735	0.026
Dose	0.945	0.819–1.089	0.433			
AFP >400	4.244	1.745–10.322	0.001	5.705	2.228–14,606	0.001
Cirrhosis	0.939	0.330–2.673	0.906			
LSMM	3.085	1.464–6.504	0.003	3.365	1.490–7.596	0.002

Variables with *p* < 0.05 in the univariate analysis were included in a multivariate Cox regression analysis with forward selection. In the multivariate analysis only values for significant results are given.

1 0/A = 0, B/C = 1.
